# Factors influencing protective behavior in the post-COVID-19 period in China: a cross-sectional study

**DOI:** 10.1186/s12199-021-01015-2

**Published:** 2021-09-23

**Authors:** Guiqian Shi, Xiaoni Zhong, Wei He, Hui Liu, Xiaoyan Liu, Mingzhu Ma

**Affiliations:** 1 Department of Epidemiology and Health Statistics, School of Public Health and Management, Chongqing Medical University, Chongqing, China; 2 Research Center for Medicine and Social Development, Chongqing Medical University, Chongqing, China; 3 Innovation Center for Social Risk Governance in Health, Chongqing Medical University, Chongqing, China

**Keywords:** Protective behavior, Model, COVID-19, Post-pandemic, Structural equation modeling, Cross-sectional study

## Abstract

**Background:**

The study aimed to explore the factors influencing protective behavior and its association with factors during the post-COVID-19 period in China based on the risk perception emotion model and the protective action decision model (PADM).

**Methods:**

A total of 2830 valid questionnaires were collected as data for empirical analysis via network sampling in China. Structural equation modeling (SEM) was performed to explore the relationships between the latent variables.

**Results:**

SEM indicated that social emotion significantly positively affected protective behavior and intention. Protective behavioral intention had significant direct effects on protective behavior, and the direct effects were also the largest. Government trust did not have a significant effect on protective behavior but did have a significant indirect effect. Moreover, it was found that government trust had the greatest direct effect on social emotion. In addition, we found that excessive risk perception level may directly reduce people’s intention and frequency of engaging in protective behavior, which was not conducive to positive, protective behavior.

**Conclusion:**

In the post-COVID-19 period, theoretical framework constructed in this study can be used to evaluate people’s protective behavior. The government should strengthen its information-sharing and interaction with the public, enhance people’s trust in the government, create a positive social mood, appropriately regulate people's risk perception, and, finally, maintain a positive attitude and intent of protection.

## Introduction

Coronavirus disease 19 (COVID-19) has become a major public health emergency of international concern since its first outbreak in late December 2019. The virus outbreak was declared a global pandemic by the World Health Organization (WHO) on March 11, 2020, and the situation remains very serious [1]. The prevention and control of the pandemic urgently require the active participation of governments, health authorities, researchers, and the general public [2]. Most COVID-19 patients have mild symptoms, but some develop serious complications (acute respiratory distress syndrome, myocardial damage, renal insufficiency, etc.), and even after treatment, there can be unimaginable sequelae (heart failure, chronic kidney disease, psychological problems, etc.) [3, 4]. As of June 29, 2021, there were 181.17 million confirmed cases of COVID-19 worldwide, including more than 3.93 million deaths, and the number of new confirmed cases daily was 309048 [5]. Studies have shown that COVID-19 is highly infectious and lethal, significantly more so than SARS-CoV (8098 infected cases and 774 deaths) and MERS-CoV (2494 infected cases and 858 deaths) [6]. As a new infectious disease, our understanding of its natural host and intermediate host is still unclear. The incubation period of COVID-19 can be as long as 24 days and infectious to a certain extent. Moreover, the routes of transmission are diversified. The abovementioned factors pose enormous challenges to the contention of the spread of COVID-19 [3, 7, 8].

Facing the global COVID-19 pandemic, different protective measures have been taken to prevent the further outbreak and spread of the pandemic. Community isolation and closed management have been performed by governments in several countries, such as Bangladesh [9], Serbia [10], Italy [11], South Africa [12], and the Philippines [13]. Furthermore, governments and experts in the prevention and control of infectious diseases have carried out health education and behavioral guidance through various channels [13]. Currently, there is no specific treatment for COVID-19. The process of vaccinating the public against COVID-19 is being sped up in China, but the challenge to achieve herd immunity is tremendous, and the issue of immune persistence has not yet been determined. Importantly, experts recommend maintaining personal protection even for those who have been vaccinated. Therefore, individual protective behavior remains the most immediate and most powerful weapon for reducing COVID-19 infection rates.

In the context of the COVID-19 globalization crisis, China has entered the stage of normal prevention and control since April 29, 2020, which is considered the post-pandemic period [14]. In this period, China’s effective strategy for controlling the outbreak and spread of the pandemic is to prevent “import from outside and rebound from inside.” Anti-infection and anti-rebound have become the top priorities, and this situation is likely to last for a long time. Therefore, in addition to government guidance and efforts, it is important that people continue to adopt protective behavior in the post-pandemic period. According to numerous previous studies, effective measures to reduce COVID-19 infection include isolation at home, keeping a social distance, wearing masks outside, observing personal hygiene, and avoiding crowds [10, 15]. With regard to behavioral decision-making, the public can directly decide whether to adopt protective behavior or not. Previous researchers have also provided a large number of studies on the various factors affecting protective behavior, but the current situation in China reflects a lack of targeted guidance and suggestions. Therefore, it is necessary to understand how public protection behavior in China has emerged in the post-pandemic period. Accordingly, the potential predictors of people’s protective behaviors should be further clarified, so that targeted interventions can be proposed to enhance people’s adherence to the recommended protective behavior.

In the post-COVID-19 pandemic period, this risk-prevention behavior needs to be a part of people’s daily lives and work habits to reduce their own risk of infection. Therefore, based on the risk perception emotion model and protective action decision model (PADM), this study explored the factors that affect people’s protective behavior in the post-pandemic period and puts forward scientific and effective guidance suggestions, which is of great practical significance to the prevention and control of the epidemic in the post-pandemic period.

## Theoretical framework

**Fig. 1:**
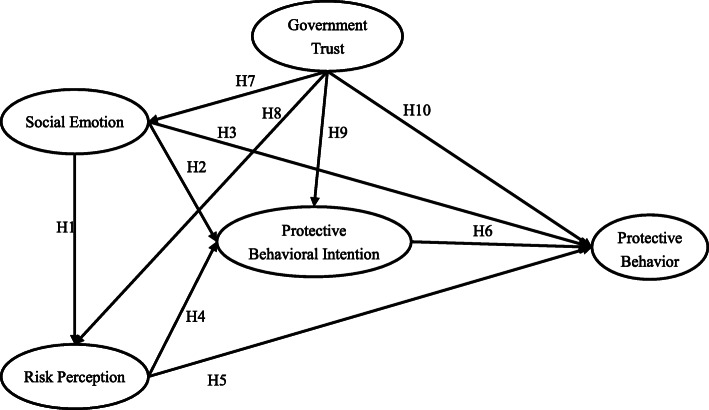
Theoretical framework

Figure 1 represents the theoretical framework constructed in this study was based on the risk perception emotional model and the PADM. Despite its success in predicting behavior, the research added the factor of government trust, combined with the current situation, to improve our ability to predict behavior in the post-COVID-19 pandemic period.

Previous studies indicated that emotional states could reflect the potential of the individual psychological structure, including anger, fear, anxiety, and emotional decision-making [16]. Social emotion is the externalization of personal emotions and also serves as social signals of all of one’s emotions to others. It can involve subjective norms, social influence, and social rules while also highlighting the importance of certain relationships and helping to maintain and restore those relationships when needed, thus acting as a social regulator [17]. Therefore, social emotion will become the driving force for behavioral decisions in social information communication. According to the risk perception emotion model, social emotion is defined as the individual's subjective evaluation and psychological feelings regarding their current social environment. The model holds that emotion-based perceived susceptibility is an important variable that causes individuals to produce behavioral responses. In the face of specific harm or threat, changing the corresponding affective association can effectively change the behavioral response [18]. Consistent with the Assessment Trend Framework (ATF) study [19], Lanciano et al. [20] found that different emotional states increase different risk perceptions. Besides, in previous studies on infectious diseases, such as SARS, empirical results showed that there was a direct relationship between emotion and behavior [21, 22]. However, some scholars have proposed that different emotional states have different mechanisms of influence on behavior. For example, anxiety often comes from the uncertainty of threats, which drives individuals to seek out more information to reduce their inner anxiety [23], while fear usually stimulates or changes one’s own behavior to solve or even avoid problems. Of course, excessive fear may also cause individual avoidance behavior [24]. In addition, behavioral intention was considered to be the closest determinant of behavior in many major theories [25]. Emotion, as a mediating variable or a regulating variable, can directly affect behavioral intention, which has been proved in many fields [26–28]. Finally, Zhong et al. [29] emphasized the importance of encouraging a clear understanding of real risks and developing mitigation strategies while taking into account people’s emotional and mental health. Thus, based on the above discussion, we propose the following hypotheses:

- H1. Social emotion has a significant direct impact on risk perception.
- H2. Social emotion significantly and directly affects individuals’ protective behavioral intention.
- H3. Social emotion has a significant and direct influence on individual protective behavior.

Risk perception is a common factor in the risk perception emotion model and the PADM. It is based on the cognition of surrounding risks and reflects people’s direct judgment or subjective prediction of various risks that may occur, such as a dangerous event [18, 30]. Risk perception is divided into individual and social aspects [31]. The former is used to assess one’s own possible risk of infection; the latter is used to assess the possible risk of infection for other people or society as a whole. The decision to engage in protective behavior (protective behavioral intention) is the subjective evaluation of whether an individual is willing to carry out adaptive behavior [32]. Based on the PADM, risk perception indirectly affects individuals’ protective behavior through their behavioral intention. This relationship has been confirmed in many studies on health-related behaviors, and it is believed that risk perception and behavioral intention are important factors for predicting individual adaptive behavior [13, 25, 33, 34]. Furthermore, the PADM in the subsequent expansion reveals that individual risk perception is not a complete intermediary but rather a partial intermediary, which provides evidence for the direct relationship between risk perception and behavior [35]. Existing research shows that the relationship between risk perception and behavior is still unclear. Most scholars believed that there is a positive correlation between the two, meaning that when people were aware of their probability or susceptibility of getting infected with a disease, they stimulated their own behavioral responses and were more likely to comply with various relevant recommended behaviors [36–38]. However, Hay et al. [39] believed that there was a two-way relationship between risk perception and behavior, meaning people who perceived a higher risk for themselves would influence other individuals, who engaged in risky behaviors, to make behavioral changes, and risk perception would be reduced once protective behaviors were adopted. At the same time, Aerts et al. [40] proposed that it was crucial to promote the generation of adaptive behaviors by improving people’s risk perception so that the reduction of risk perception would not translate into lower adaptive behaviors. In addition, Wachinger et al. [41] proposed the “risk perception paradox,” arguing that higher risk perception did not necessarily lead to behavioral preparation, nor did it necessarily reduce people’s risky behaviors. Then, what is the relationship between risk perception and behavior in the post-pandemic period? We propose the following hypotheses:

- H4. Risk perception has a significant direct impact on protective behavioral intention.
- H5. Risk perception directly affects the individual's protective behavior.
- H6. Protective behavioral intention directly affects the individual's protective behavior.

In the case of public health emergencies, the government bears the main responsibility for management, prevention, and control, and it is a force that cannot be ignored in emergency management. Early warning, timely monitoring, and rapid prevention and control of the pandemic cannot be achieved without correct government management and guidance. In turn, complying with recommended behavior is dependent upon the government’s epidemic guidelines. In this study, government trust is defined as individuals’ recognition and trust assessment of the government’s crisis management, including information-sharing and pandemic prevention and control [42]. The former mainly means the timely publication of accurate pandemic-related information and continuous publicity of protection information. The latter mainly refers to stopping the spread of the pandemic and providing disease relief. Research in other areas found that trusting policies can also be achieved through people’s emotional responses. The authors believe that irrational actions based on emotional judgments are more likely to be due to people’s lack of knowledge or experience about new, unknown, and ambiguous events [43]. In the case of COVID-19, many researchers have found that government trust affects people’s risk perceptions, protective behaviors, and behavioral intentions [44, 45]. When government trust is high, people believe that various control measures and the information provided by the government are correct and unbiased, which will reduce people’s risk perception level and make them more willing to comply with the protective behaviors recommended by the government. Therefore, we propose the following hypotheses:

- H7: Government trust significantly affects social emotion.
- H8: Government trust significantly affects individuals’ risk perceptions.
- H9: Government trust significantly affects individuals’ protective behavioral intentions.
- H10: Government trust significantly affects individuals’ protective behaviors.

## Methods

### Participants

A cross-sectional survey was utilized in this study. Participants were recruited in China from February 1 to February 20, 2021. Before the survey began, we provided an informed consent form, and the questionnaire could only be filled out after the participants had read and agreed to it. Inclusion criteria are as follows: (1) age ≥15 years old, (2) basic reading and writing skills and the ability to use electronic devices, and (3) agreed to participate in this survey. Exclusion criteria are as follows: (1) currently or previously infected with COVID-19 and (2) questionnaire completion times of <0.5 min or > 30 min. Finally, a total of 3437 participants participated in this study.

### Data collection

All participants could complete an anonymous online questionnaire (Questionstar: a professional online survey tool). In order to ensure the quality of the questionnaire, we implemented a strict screening mechanism. In the online questionnaire, each question was set as a required item, each participant could only submit it once according to their IP and WeChat ID, and the filling time was monitored in real-time to exclude invalid questionnaires. In addition, the researchers carried out a final quality audit, and participants who passed the audit were given a reward of 5 RMB. Of the 3437 participants, 2830 valid questionnaires were screened systematically and manually, with an availability rate of 82.34%.

### Questionnaire

Following the theoretical framework and combining experts’ advice and preliminary survey results, the relevant items were revised and optimized. A formal questionnaire was finally developed and determined to assess the relationship between the public’s risk perception and protective behavior and the potential factors influencing its behavior in the post-pandemic period. The questionnaire consists of six parts: (1) sociodemographic information, including sex, age, residence, province of residence, ethnicity, educational background, marital status, employment status, self-rated economic status, chronic illness, and self-rated health status; (2) social emotion (SE), based on Hareli and Parkinsion [17]; (3) risk perception (RP), based on the WHO [46] and Tyler et al. [31]; (4) protective behavioral intention (PBI), derived from the study of Ajzen et al. [47]; (5) protective behavior (PB), based on a revision of personal hygiene and protection recommendations of the WHO [46] and the State Council of the People’s Republic of China [48]; and (6) government trust, based on studies by Azadi et al. [49] and Samadipour et al. [50]. Each construct contained in the SEM was evaluated using a 5-point Likert scale. Except for protective behavior, for which the scale ranged from 1 = not at all to 5 = very much so, constructs were measured on a scale from 1 = strongly disagree to 5 = strongly agree. Upon completion of the above information, participants terminated the survey. Each construct and its corresponding items in the study are presented in Table 1.

**Table 1:** Constructs and corresponding items in the research

Constructs	Items	Measures
Social emotion (SE)	SE1	It is important to be protected against COVID-19 at all times
	SE2	The real-time push of COVID-19 information makes me pay attention
Risk perception (RP)	RP1	I think I may be likely to get infected with COVID-19
	RP2	I think I may be more susceptible to COVID-19
	RP3	I think someone around me may be infected with COVID-19
Protective behavioral intention (PBI)	PBI1	I would like/continue to take precautions against COVID-19
	PBI2	I will implement COVID-19 preventive actions more frequently in the future
	PBI3	I would try more effective measures to prevent COVID-19
Protective behavior (PB)	PB1	Wash my hands regularly and maintain hand hygiene
	PB2	Cover myself when I cough or sneeze
	PB3	Wear masks correctly in confined spaces/crowded areas
	PB4	Clean/disinfect frequently touched surfaces such as door handles, railings
Government trust (GT)	GT1	I think the government has provided real information about COVID-19
	GT2	I think the government has provided adequate information about COVID-19 protection
	GT3	I think the government responded quickly to COVID-19

According to relevant theories and previous studies, the protective behavior of COVID-19 may be affected by gender, age, ethnicity, and self-rated economic status [51–53]. Therefore, this study contained 4 control variables that might affect the protective behavior of COVID-19; these included gender, age, ethnicity, and self-rated economic status. We set a dummy variable for gender, with 1 for male and 2 for female. We used a discrete variable for the ages, with age = 1, 2, 3, 4, 5, and 6 for age under 20, between 21 and 30, between 31 and 40, between 41 and 50, between 51 and 60, and over 61 years, respectively. We used a dummy variable for ethnicity, with 1 for Han nationality and 2 for ethnic minorities. Finally, we used a discrete variable to assess self-rated economic status of our participants with self-rated economic status = 1, 2, 3, 4, and 5 for very bad, bad, general, good, and very good, respectively.

### Statistical analysis

The statistical analysis was performed using IBM SPSS 25 and AMOS 24. The mean, standard deviation, and extreme value were used for the statistical description of measurement data, while frequencies and percentages were used for the statistical description of enumeration data. Confirmatory factor analysis (CFA) was used to evaluate the relationship between the measured and the latent variables. The maximum likelihood estimation method was used to conduct SEM modeling in AMOS 24 software to evaluate the theoretical model of this study. The overall model fitting evaluation indexes and criteria were as follows: chi-square degree of freedom ratio (*χ*^2^/DF ratio) < 5.0 [54], root mean square error of approximate (RMSEA) < 0.05, incremental fit index (IFI) > 0.90, goodness of fit index (GFI) > 0.90, adjusted goodness of fit index (AGFI) > 0.90, Tucker-Lewis index (TLI) > 0.90, and comparative fit index (CFI) > 0.90 [55–57].

## Results

### General characteristics of participants

Two thousand eight hundred thirty participants aged 15–83 years (mean=36.64, standard deviation=14.06) were included in the study. The sociodemographic descriptive statistics of the participants are shown in Table 2, indicating that 61.20% of participants were women. Most of the participants were between 21 and 30 years old (33.82%), had an urban residence (61.59%), lived in Chongqing municipality (49.36%), were of Han nationality (96.86%), had obtained a bachelor’s degree or above (40.60%), were married (59.65%), were employed (56.64%), had an average economic status (62.47%), had a good health status (44.98%), and no chronic illnesses (92.05%).

**Table 2:** Sociodemographic descriptive statistics of participants (*N*=2830)

Characteristics	Category	Frequency	Percentage (%)
Gender	Male	1098	38.80
	Female	1732	61.20
Age(years)	Under 20	279	9.86
	21–30	957	33.82
	31–40	508	17.95
	41–50	553	19.54
	51–60	390	13.78
	61 and above	139	4.91
	Missing ^a^	4	0.14
Residence	Urban	1743	61.59
	Rural	1087	38.41
Province of residence	Chongqing Municipality	1397	49.36
	Shanxi Provinces	322	11.38
	Sichuan Provinces	318	11.24
	Hebei Provinces	147	5.19
	Shandong Provinces	133	4.70
	Others	513	18.13
Ethnicity	Han	2741	96.86
	Ethnic minorities	89	3.14
Education background	Elementary school or below	78	2.76
	Junior high school	412	14.56
	Senior high school/Vocational high school/Technical secondary school	596	21.06
	Junior college	595	21.02
	Bachelor’s degree or above	1149	40.60
Marital status	Unmarried	1018	35.97
	Married	1688	59.65
	Divorced	105	3.71
	Widowed	19	0.67
Employment status	Employed	1603	56.64
	Retirement	296	10.46
	Internal student	560	19.79
	Jobless	371	13.11
Self-rated economic status	Very bad	105	3.71
	Bad	358	12.65
	General	1768	62.47
	Good	439	15.51
	Very good	160	5.65
Chronic illness	Yes	225	7.95
	No	2605	92.05
Self-rated health	Very bad	25	0.88
	Bad	55	1.94
	General	707	24.98
	Good	1273	44.98
	Very good	770	27.21

^a^ Missing values were due to incorrect filling

### Measurement model analysis

We first conducted a CFA to evaluate the validity of the scale. In the measurement model, after the deletion of item PB4, the overall measurement model fitted well. The *χ*^2^/DF value was 4.527, which was below the critical value of 5.0. The RMSEA value was 0.035, which was clearly lower than 0.5. The IFI, GFI, AGFI, TLI, and CFI values were 0.990, 0.984, 0.975, 0.986, and 0.990, respectively, which were all greater than the recommended cutoff value of 0.9. These results indicated that the measurement model had good structural validity. The unstandardized factor loadings were significant (*P* < 0.001). As presented in Table 3, the standardized factor loadings ranged from 0.709 to 0.934, which were clearly all greater than the 0.50 cutoff value. The AVE values ranged from 0.517 to 0.794, also all greater than 0.50. The CR values ranged from 0.762 to 0.920, which were all greater than the cutoff value of 0.70. These results confirmed that the measurement model had good convergence validity [58, 59]. As shown in Table 4, the square roots of AVE were all higher than the correlation coefficients between constructs, indicating that the measurement model had good discriminative validity. Moreover, all constructs in this study were extracted on the basis of previous research theories and modified and supplemented in combination with relevant characteristics of this study and expert opinions, which ensured good content validity of the questionnaire.

**Table 3:** Factor loadings of influence factors by measurement model test

Constructs	Items	Factor loadings	Cronbach’s α	CR	AVE
Social emotion (SE)	SE1	0.826	0.805	0.805	0.674
	SE2	0.816			
Risk perception (RP)	RP1	0.844	0.831	0.833	0.626
	RP2	0.807			
	RP3	0.717			
Protective behavioral intention (PBI)	PBI1	0.881	0.919	0.920	0.794
	PBI2	0.857			
	PBI3	0.934			
Protective behavior (PB)	PB1	0.726	0.762	0.762	0.517
	PB2	0.709			
	PB3	0.721			
Government trust (GT)	GT1	0.859	0.911	0.912	0.775
	GT2	0.924			
	GT3	0.856			

Factor loadings, standardized factor loadings

*CR* composite reliability, *AVE* average variance extracted

**Table 4:** Means, standard deviations, correlations, and square root of AVE (bold font) among model constructs

Constructs	Mean	SD	SE	RP	PBI	PB	GT
SE	4.38	0.68	**(0.821)**				
RP	1.85	0.72	−0.106***	*(0.791)*			
PBI	4.48	0.61	0.563***	−0.191***	*(0.891)*		
PB	4.35	0.60	0.514***	−0.249***	0.697***	*(0.719)*	
GT	4.29	0.65	0.590***	−0.150***	0.442***	0.367***	*(0.880)*

*SD* standard deviations, *AVE* average variance extracted. The bold font is square root of AVE

***p < 0.001

Finally, Cronbach’s α was utilized to measure the reliability of the measurement model. The Cronbach α values of each construct were all greater than 0.7 (see Table 3), indicating that the internal consistency of the questionnaire was high and that the reliability was also good.

### Structural model analysis and hypothesis testing

First, AMOS software was used to conduct the SEM fitting test. The overall adaptability of the model to the sample data was good: *χ*^2^/DF= 4.964, RMSEA= 0.037, IFI= 0.979, GFI= 0.977, AGFI= 0.967, TLI= 0.974, and CFI= 0.979. In the structural model, the *R*^2^ of PB was 0.525, which was higher than the critical value of medium explanatory power (0.33) [60], showing that all of the variables in the model could explain 52.5% of the PB variation. Therefore, the structural model in this study had a strong explanatory ability and the established model was good.

**Fig. 2:**
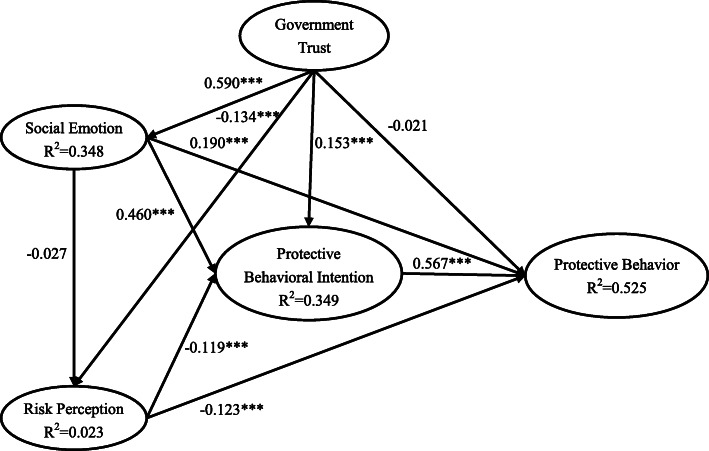
Estimation of the model of protective behavior. Note: ^*^p<0.05, ^**^p<0.01, ^***^p<0.001.

Next, Fig. 2 shows an estimation of the model for evaluating people's protective behavior during the post-COVID-19 period in China. The results showed that SE was positively associated with PBI (*β*=0.460, *T*=17.474, *P* < 0.001) and PB (*β*=0.190, *T*=6.527, *P* < 0.001). RP had significant direct effects on PBI (*β*=−0.119, *T*=−6.429, *P* < 0.001) and PB (*β*=−0.123, *T*=−6.317, *P* < 0.001). PBI also positively affected PB (*β*=0.567, *T*=22.023, *P* < 0.001). However, SE had no effect on RP (*β*=−0.027, ns). Thus, hypotheses 2, 3, 4, 5, and 6 were all supported, while hypothesis 1 was not supported. The results showed that the effects of GT on SE (*β*=0.590, *T*=27.577, *P* < 0.001), RP (*β*=−0.134, *T*=−4.722, *P* < 0.001), and PBI (*β*=0.153, *T*=6.481, *P* < 0.001) were significant. However, the effect of GT on PB was not significant (*β*=−0.021, ns). Thus, hypotheses 7, 8, and 9 were supported, while hypothesis 10 was not supported. In summary, the empirical results of our study supported eight hypotheses and did not support two hypotheses (H1 and H10).

Finally, a bootstrapping test was utilized to estimate the unstandardized coefficients and significance levels of indirect effects. In total, 5000 bootstrap samples were tested, and the significance level was determined by the percentile method (95% percentile confidence level). The results regarding the direct, indirect, and total effects of the structural model are shown in Table 5. In conclusion, the total effects of SE, RP, PBI, and GT on PB were 0.415, −0.138, 0.558, and 0.304, respectively.

**Table 5:** Direct effects, indirect effects, and total effects of structural model

Path	Direct effects	*p* value	Indirect effects	*p* value	Total effects	*p* value
SE → RP	−0.034	0.372			−0.034	0.372
SE → PBI	0.425	0.001	0.003	0.372	0.428	0.001
SE → PB	0.173	0.001	0.242	0.001	0.415	0.001
RP → PBI	−0.088	0.001			−0.088	0.001
RP → PB	−0.089	0.001	−0.049	0.001	−0.138	0.001
PBI → PB	0.558	0.001			0.558	0.001
GT → SE	0.548	0.001			0.548	0.001
GT → RP	−0.156	0.001	−0.019	0.372	−0.175	0.001
GT → PBI	0.132	0.001	0.248	0.001	0.380	0.001
GT → PB	−0.018	0.449	0.322	0.001	0.304	0.001

The above results are unstandardized values

## Discussion

First of all, the risk perception emotion model and the PADM were used for reference. At the same time, in order to improve the prediction effect of behavior, we added the factor of government trust to construct the theoretical framework of this study. Next, a total of 2,830 questionnaires were collected via an online survey. Finally, SEM was used to analyze and conduct hypothesis testing of the relationships among social emotion, risk perception, protective behavioral intention, protective behavior, and government trust so as to evaluate the factors influencing people’s protective behavior in the post-COVID-19 pandemic period.

The SEM results showed that social emotion had a direct positive effect on people’s protective behavior and willingness to engage in it. It can be concluded that the overall social emotion of people in the post-pandemic period was good, which had a positive impact on their protective decision-making and behavior and also meant that better social emotion helped people form and maintain a positive attitude toward protective measures. Individuals’ emotions and psychological cognition will be affected by external information and the people around them, which can stimulate their understanding and perception of the current pandemic to a certain extent [61]. Interestingly, social emotion had no direct effect on risk perception. In other words, China’s pandemic prevention and control have entered the post-pandemic stage, and positive social emotion has not significantly stimulated people's risk perception. One possible reason for this observation was that people believed that the current social environment was good and that the infection risk was relatively low. However, considering that the epidemic had not completely disappeared and that it may occur again, people had not completely eliminated their worries, anxiety, or panic about the pandemic and still maintained a certain level of risk perception [62]. Therefore, better social emotion had no significant impact on people’s risk perception.

Risk perception was negatively correlated with protective behavior and willingness to engage in it, which is consistent with Guo et al.’s findings [34]. This meant that excessive risk perception was not conducive to the adoption of recommended adaptive behaviors and may produce non-recommended behaviors or negative behaviors [63]. It can also be argued that those with high level of trust in the government were most likely to believe that their living environment was relatively safe despite a rebound of the pandemic, that their risk of contracting the disease was low or that they were less susceptible to the pandemic, and that their level of risk perception was weaker than they were during the COVID-19 pandemic period [6]. Therefore, it is necessary for relevant departments to guide individuals to maintain a moderate level of risk perception and trust the government to adopt active protective behavior.

The behavioral intention variable is considered to be the best predictor of individuals’ compliance with the recommended behavior [64]. The strength of behavioral willingness is closely related to whether people are willing to cooperate and how closely they cooperate. In other words, long-term, effective compliance with the recommended protective behavior depends on people's willingness to engage in such behavior. The SEM showed that protective behavioral intention could directly and positively influences people’s protective behavior, which is consistent with many prior research results [13, 65]. The results suggested that behavioral intention was the most direct and most important factor influencing people’s protective behavior in the post-COVID-19 pandemic period. Hence, in order to better guide people to follow the official recommended protective behaviors, the government should give full consideration to people’s willingness to engage in these and enhance people’s cooperation, which can promote the effectiveness and sustainability of the implementation of these behaviors to a certain extent and can also contribute to the formation of better hygiene habits in daily life.

The study found that the direct effect of government trust on social emotion, risk perception, and willingness to engage in protective behavior was significant. Government trust was not directly related to protective behavior, but it had a large indirect effect. It can be said that because of trust in the government, various prevention and control measures, such as COVID-19 vaccination, carried out by the government, will stimulate people’s social emotion, arouse their concern and attention regarding these events, accelerate their correct understanding and perception thereof, and reduce their fear and anxiety. Finally, it can lead people to a positive response to the pandemic [45].

We even observed that the total effects of social emotion, risk perception, willingness to engage in protective behavior, and government trust regarding protective behavior were 0.415, -0.138, 0.558, and 0.304, respectively. From what has been discussed above, our suggestion is to establish good, cooperative relations between the government and the public. On the one hand, the government should provide early warning, monitoring, and epidemic prevention and control. On the other hand, the government should do a good job with their information-sharing and interactions with the public to strengthen people’s trust in the government and create a good social atmosphere. This may lead people to maintain the correct attitude of prevention and control, reasonably responding to all kinds of emergencies.

Although this study has provided findings and recommendations, there are still some potential limitations and future directions. First, due to the influence of the pandemic, the recruitment of the survey participants was challenging, and only online recruitment could be conducted through the method of non-probability sampling. However, we collected a large number of samples to reduce deviation as much as possible. Second, we only collected data from the late period of the COVID-19 pandemic in China, and this study mainly examines the protective behavior of Chinese residents aged 15 years and above. Therefore, the application and extension of the theoretical model of this study to evaluate protective behavior regarding COVID-19 toward other countries and regions needs to be further explored. Third, due to the time lag, we adopted a cross-sectional survey design so that the dynamic changes of the influence of government trust, social sentiment, and protective behavioral intention on behavior in different periods were not tracked longitudinally.

## Conclusion

In this study, a theoretical framework was constructed based on the risk perception emotion model and the PADM. Survey data of 2830 participants were collected. This empirical study explored the factors and processes influencing people’s protective behavior in the post-COVID-19 pandemic period via SEM. The results revealed that social emotion had a significant positive effect on protective behavior and willingness to engage in them. Protective behavior intention had a direct influence on protective behavior, and this influence was the largest. Government trust had no significant positive effect on protective behavior, but it had a significant indirect effect. Moreover, we found that government trust had the greatest direct effect on social emotion. Furthermore, we observed that excessive risk perception levels might reduce people’s willingness and frequency of engaging in protective behavior, which was not conducive to positive protective behavior. Thus, based on these findings, we suggest that the government should strengthen their information-sharing and interaction with the public, enhance people’s trust in the government, create a good social emotion, properly regulate people’s risk perception, and finally, maintain a positive attitude and intent of protection.

## Data Availability

The datasets analyzed during the current study are available from the corresponding author on reasonable request.
